# Lysozyme‐induced nephropathy due to systemic granulomatous disease

**DOI:** 10.1002/ccr3.8122

**Published:** 2023-11-19

**Authors:** Hamza Ashraf, Dariusz Uczkowski, Matthew Stuart

**Affiliations:** ^1^ Overlook Medical Center Atlantic Health System Summit New Jersey USA

**Keywords:** lysozyme nephropathy, systemic granulomatous disease, sarcoidosis

## Abstract

Lysozyme‐induced nephropathy is a rare form of acute tubular injury that has almost exclusively been reported in patients with monocytic malignancies. Typically, patients will present in acute renal failure A renal biopsy is necessary to confirm the diagnosis and will demonstrate proximal tubular cells with hypereosinophilic granules, which are periodic acid‐Schiff and Jones methenamine silver‐positive. Immunohistochemical staining for lysozyme will also be present. The following rare case will describe a case of lysozyme nephropathy in a patient without any underlying hematological malignancy, but instead with systemic granulomatous disease.

## Graphical abstract

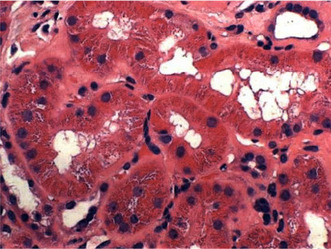

## 1 INTRODUCTION

Lysozyme, which was discovered by Alexander Fleming in 1922, is an enzyme contained primarily within monocytes and to a lesser extent in neutrophils.^1^, ^2^ The significance of lysozymes is rooted in their integral role in the innate immune response. Once the leukocytes phagocytose a bacterial pathogen, the intracellular lysozymes disturb the bacterial life cycle by hydrolyzing the bacterial cell wall and thereby facilitate death of the organism.^2^, ^3^ A superfluity of lysozyme production is sometimes seen in monocytic malignancies such as acute promyelocytic, acute monocytic, or chronic myelomonocytic leukemia (CMML). Typically, lysozymes are freely filtered by the glomerulus and reabsorbed by the proximal tubules.^4^ In rare cases, patients with monocytic malignancies can develop kidney injury secondary to a marked overproduction of lysozymes, which in turn overwhelm the absorptive capacity of the proximal tubules.^4^, ^5^ The likelihood that a patient without a monocytic malignancy will develop a lysosomal nephropathy is low, given that few disease processes garnish such a hefty lysozyme burden. Herein, we describe a case of lysosomal nephropathy in a patient without a hematological malignancy but with an anomalous finding of non‐necrotizing granulomas within the bone marrow.

## 2 CASE PRESENTATION

A 60‐year‐old male with a past medical history of heart failure with a preserved ejection fraction, severe tricuspid regurgitation, atrial fibrillation, type 2 diabetes mellitus, gout, hyperlipidemia, and chronic kidney disease presented to the emergency department with complaints of worsening shortness of breath and concomitant swelling in both of his legs for the past 2 weeks. Vital signs on presentation consisted of a heart rate of 68 beats per minute, a blood pressure of 104/72 mmHg, a respiratory rate of 17 breaths per minute, a temperature of 97.7 degrees Fahrenheit, and an oxygen saturation of 96% on room air. Physical examination was significant for an elevated jugular venous pressure with distended neck veins, a holosystolic murmur at the chest apex, decreased breath sounds, and 3+ pitting edema of the bilateral lower extremities extending up to the hips. Laboratory tests revealed a white blood cell count of 2.21/nL, a red blood cell count of 2.99/pL, a hemoglobin of 8.9 g/dL, a platelet count of 117/nL, a BUN of 49, a creatinine of 3.54 mg/dL (baseline 1.4 mg/dL), and a point of care brain natriuretic peptide level of 216 pg/mL. Additionally, the presence of moderate anisocytosis, poikilocytosis, macrocytosis, and polychromasia was detected. A flow cytometry was performed and was notable only for monocytosis. Urinalysis revealed a bland sentiment with a urine protein‐to‐creatinine ratio of 290 mg/g.

The presenting symptomology was diagnosed as having stemmed from an acute diastolic heart failure exacerbation. The patient's hospital course was uncomplicated, consisting primarily of receiving diuresis with intravenous furosemide to obtain euvolemia. The patient was discharged after resolution of his dyspnea and lower extremity edema; however, there was concern regarding his pancytopenia and persistently worsening renal function, for which the patient was told to follow up with a hematologist and a nephrologist as an outpatient. Upon discharge, patient was noted to have a creatinine of 4.32. After discharge, the patient underwent a bone marrow biopsy, which revealed a normocellular bone marrow with adequately maturing trilineage hematopoiesis and no evidence of myelodysplasia. Special stains for acid‐fast and fungal organisms (Acid‐Fast Bacillus staining and Grocott's Methenamine Silver Stain) were both negative. However, the presence of non‐necrotizing granulomas was detected in the bone marrow.

**FIGURE 1:**
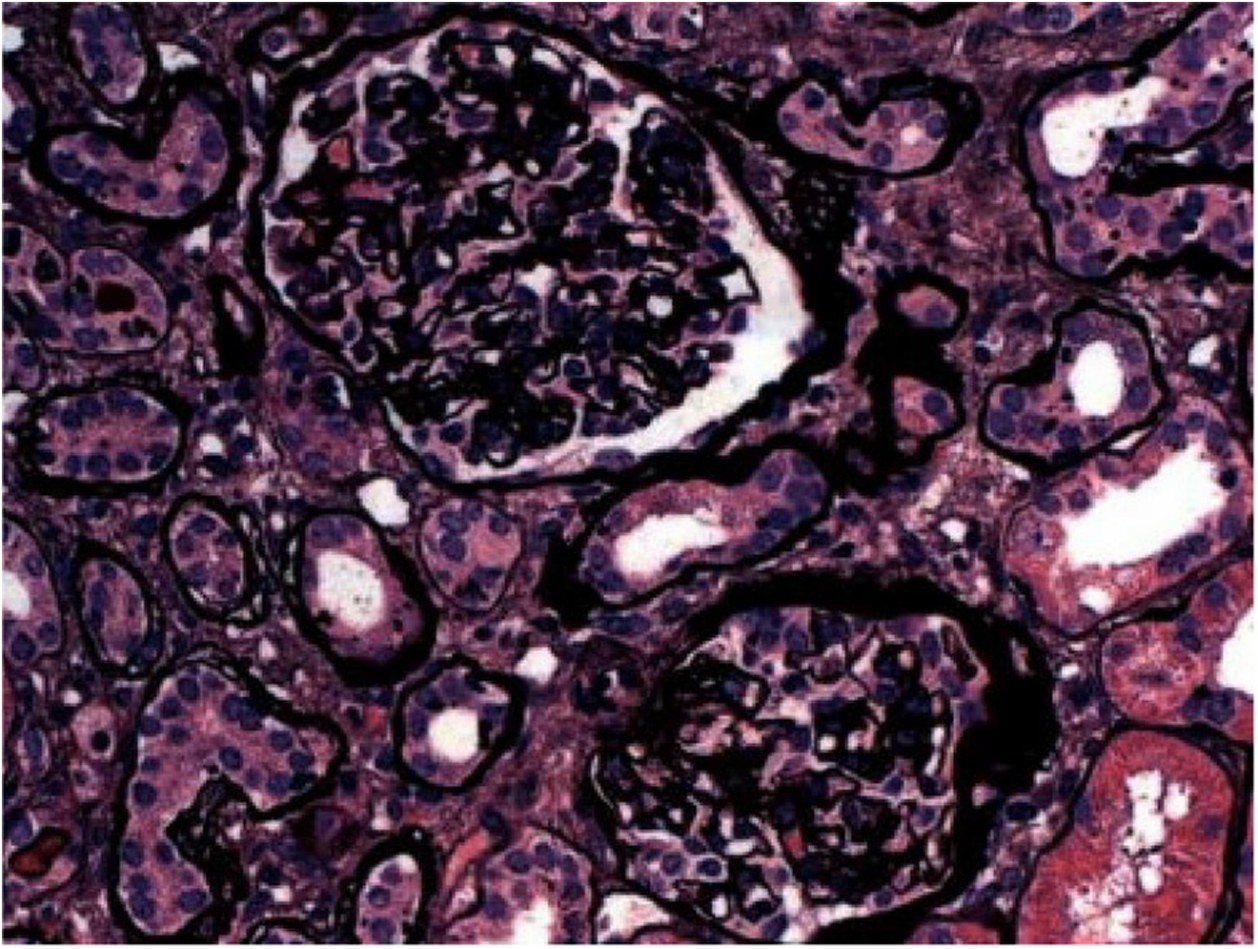
Sclerotic glomeruli with characteristic wrinkling and thickening of the glomerular basement membrane, consistent with ischemia.

**FIGURE 2:**
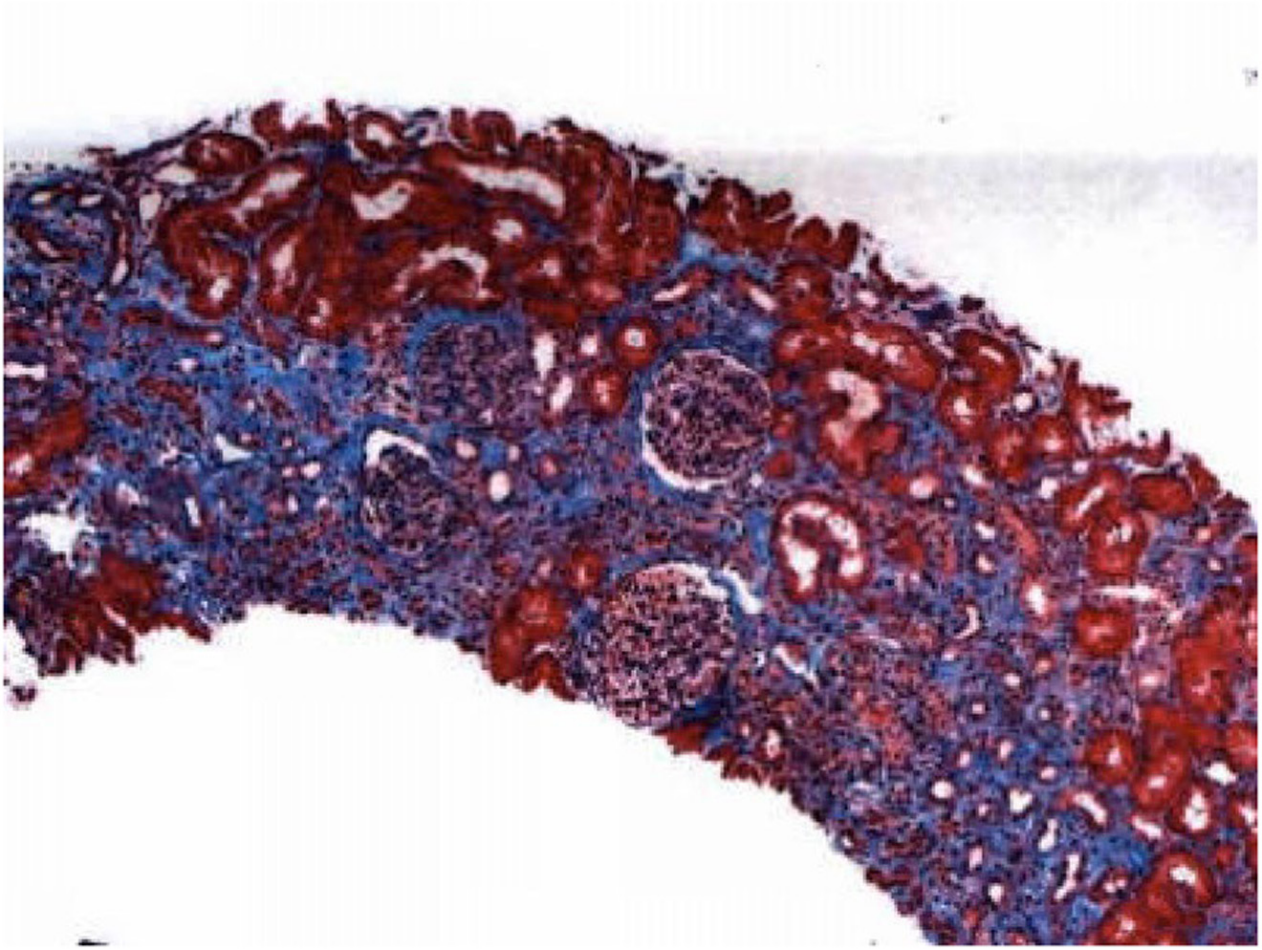
Scarred renal cortex, consistent with chronic ischemic changes.

**FIGURE 3:**
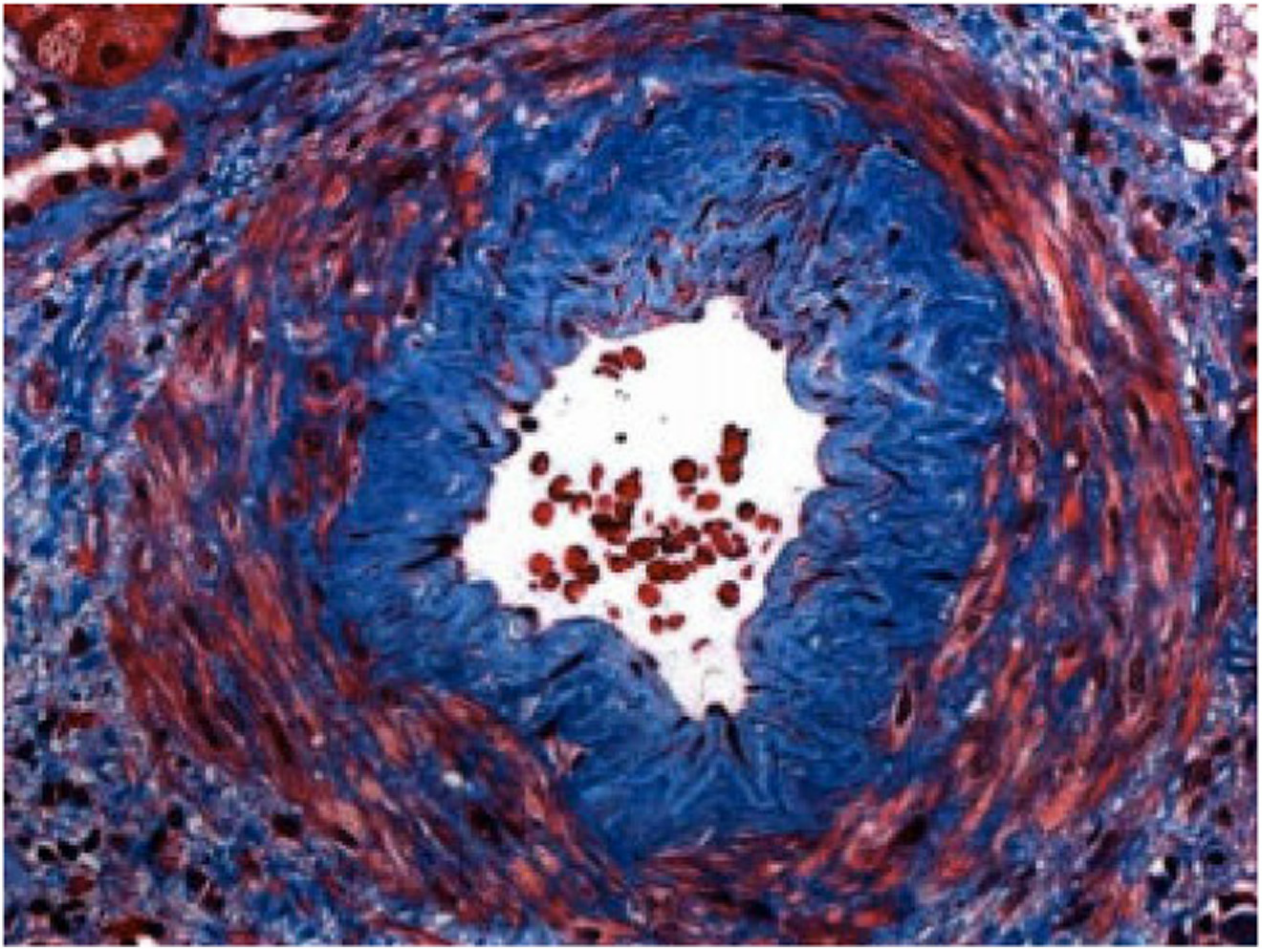
Arterionephrosclerosis.

**FIGURE 4:**
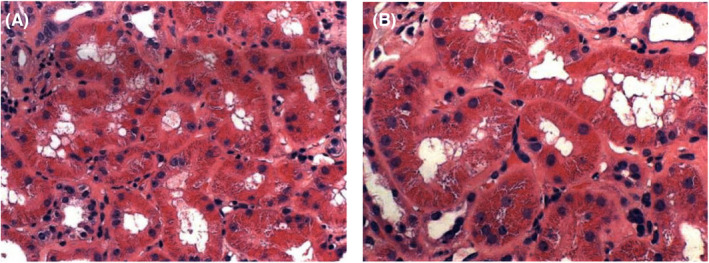
(A, B) Proximal tubules packed with granular eosinophilic protein droplets which show intense staining for lysozyme by immunochemistry.

Moreover, the patient underwent a renal biopsy, which revealed moderate tubulointerstitial scarring with focal ischemic glomerular changes and moderate to severe vascular disease, which are findings consistent with arterionephrosclerosis and chronic ischemic changes related to hypertension and heart failure, visualized in Figures 1, 2, 3. In addition, proximal tubular cells were seen engorged by refractile eosinophilic, Periodic acid–Schiff‐pale, silver‐negative resorption droplets that exhibited immunoreactivity for lysozyme, as seen in Figure 4A,B. Further outpatient workup included an elevated serum lysosome of 18.4 μg/mL, with normal serum levels of Vitamin D 1,25, angiotensin‐converting enzyme 34 U/L, and negative anti‐neutrophil cytoplasmic antibodies. The Hepatitis B surface antigen was nonreactive, and the hepatitis B core total antibody was nonreactive.

The amalgamation of findings raised suspicion for a systemic granulomatous process, and patient was started on oral steroid therapy with return of his creatinine to baseline and resolution of his pancytopenia.

## 3 DISCUSSION

As a general principle, discovering the underlying etiology of any deterioration of renal function is essential in both preventing progression of disease and in promoting recovery. In the aforementioned case, a renal biopsy demonstrated the presence of an excess of lysozymes inside the proximal tubule epithelial cells in the setting of concomitant chronic hypertensive ischemic renal disease. Lysozyme, also referred to as muramidase, is a cationic protein that is only approximately 15 kilodaltons in size and is freely filtered by the glomerulus and subsequently reabsorbed by the proximal convoluted tubule via endocytosis, and then degraded in phagolysosomes.^4^, ^6^, ^7^ Once the lysosomal burden is high enough in so that it surpasses the absorptive capacity of the proximal tubules, resulting tubular injury occurs, which can manifest as tubular necrosis, tubular atrophy, or interstitial fibrosis.^7^, ^8^

CMML and hematological malignancies with myelomonocytic differentiation are the most commonly recognized causes of lysozyme nephropathy.^6^ Theoretically, this is due to a clonal monocytic proliferation, which in turn is responsible for an abundance of lysozyme production that becomes toxic to the proximal tubular cells.^4^, ^6^ Our presented case is a rarity in that the patient underwent a bone marrow biopsy, which ruled out the presence of any hematological malignancy. Nevertheless, the bone marrow did demonstrate the presence of granulomatous disease. Given that the patient's interferon‐gamma release assay and his ANCA serologies have been negative, there is suspicion for systemic granulomatous disease. Sarcoidosis is certainly a possibility given its nonspecific diagnostic criteria; however, it is worth noting that serum markers for sarcoidosis, with the exception of serum lysozyme, including ACE, dihydoxyvitamin D, and calcium levels returned within normal limits for this patient.

Renal injury in patients with granulomatous disease such as sarcoidosis is rare, but when it does occur, the pathophysiology is driven commonly by calcium renal deposits and by parenchymal tubulointerstitial nephritis.^9^ In our case, although neither finding was appreciated on renal biopsy, we attribute the lysozyme nephropathy to the granulomatous disease. Given that the patient's serum lysozyme was elevated and given that serum lysozyme levels have been noted to be elevated in patients with sarcoidosis, the latter can be attributed to the former in this case.^10^, ^11^ Moreover, the patient's renal function improved significantly and returned to baseline with the initiation of steroid therapy, which also strengthens the argument that the underlying etiology in this case was the granulomatous disease. This rare case of lysozyme nephropathy secondary to granulomatous disease lends itself to being a diagnosis of exclusion and shines a light on the idea that this is a disease process that often goes undiagnosed either due to lack of a renal biopsy or lack of detection of granulomatous disease.

## AUTHOR CONTRIBUTIONS

**Hamza Ashraf:** Conceptualization; writing – original draft; writing – review and editing. **Dariusz Uczkowski:** Conceptualization; writing – original draft; writing – review and editing. **Matthew Stuart:** Supervision.

## FUNDING INFORMATION

No financial support was necessary for the preparation of this manuscript or acquiring data.

## CONFLICT OF INTEREST STATEMENT

All authors declare: no support from any organization for the submitted work; no financial relationships with any organizations that might have an interest in the submitted work in the previous 3 years; and no other relationships or activities that could appear to have influenced the submitted work.

## CONSENT

Written informed consent was obtained from the patient to publish this report in accordance with the journal's patient consent policy.

## Data Availability

All data underlying the results are available as part of the article and no additional source data is required.
